# Myocarditis and myositis/myasthenia gravis overlap syndrome induced by immune checkpoint inhibitor followed by esophageal hiatal hernia: A case report and review of the literature

**DOI:** 10.3389/fmed.2022.950801

**Published:** 2022-11-15

**Authors:** Beibei Yin, Junjuan Xiao, Xuan Wang, Xingyu Li, Yaping Guan, Jinghua Chen, Pengxi Han, Kun Li, Jun Wang

**Affiliations:** ^1^Department of Oncology, The First Affiliated Hospital of Shandong First Medical University and Shandong Provincial Qianfoshan Hospital, Jinan, China; ^2^Shandong Key Laboratory of Rheumatic Disease and Translational Medicine, Jinan, China; ^3^Shandong Lung Cancer Institute, Jinan, China; ^4^Department of Radiology, The First Affiliated Hospital of Shandong First Medical University and Shandong Provincial Qianfoshan Hospital, Jinan, China; ^5^Shandong Provincial Key Laboratory of Medical and Health Abdominal Imaging, Jinan, China; ^6^Shandong Institute of Neuroimmunology, Jinan, China; ^7^Department of Gastroenterology, The First Affiliated Hospital of Shandong First Medical University and Shandong Provincial Qianfoshan Hospital, Jinan, China

**Keywords:** PD-1 inhibitor, esophageal hiatal hernia, myositis, immune-related adverse event, myocarditis, myasthenia

## Abstract

Immunotherapy with programmed death 1 (PD-1) inhibitor has shown activity as first- or second-line treatment for various metastatic human malignancies. Immune-related adverse events (irAEs) are now well-described, and most organ sites are potentially influenced, but the prevalence of myocarditis and myositis/myasthenia gravis (MG) overlap syndrome following esophageal hiatal hernia induced by immunotherapy is rarely reported. Here, we describe a 71-year-old woman with a progressed unresectable extrahepatic cholangiocarcinoma and biliary obstruction. She had no prior history of muscle weakness and neuromuscular disease with a normal body mass index. She was treated with sintilimab as a rescue regimen of immunotherapy. After the first cycle of treatment, she experienced a grade 4 myopathy including simultaneous myositis, myalgia, and myocarditis due to multiple injuries in her cardiac, skeletal, and ocular muscles. She had elevated levels of creatine kinase (CK), cardiac troponin I, and myoglobin (MYO), but MG and myositis-specific and myositis-related antibodies were negative. Immunotherapy was discontinued and pulse high-dose methylprednisolone with a slow tapering and intravenous immunoglobulin (IVIG) was initiated. Two weeks later, the patient’s clinical presentation improved significantly. A subsequent cardiac magnetic resonance (MR) examination revealed an old myocardial injury that may be a result of immune-related cardiac toxicity. In the third month following the PD-1 inhibitor therapy, she restarted systemic chemotherapy in combination with an anti-angiogenic agent but without immunotherapy. Half a year later, she complained of repeated abdominal distension and radiographic examinations and endoscopy showed a clinically confirmed diagnosis of sliding hiatal hernia of the esophagus and gastroesophageal reflux disease. Due to mild symptoms associated with gastroesophageal reflux, she was suggested close monitoring with acid secretion blockade rather than immediate surgical intervention. The severity for patients with myositis and myocarditis accompanied without MG is similar to those with MG. Considering the use of PD-1 inhibitors is increasing in cancer patients, physicians should therefore pay more attention to immunotherapy-induced myocarditis with myositis/MG overlap syndrome. Since we hypothesize diaphragmatic hiatal hernia as a potential consequence of immunotherapy-induced myositis, reports on hiatal hernias subsequent to immunotherapy-induced myositis are needed.

## Introduction

As a novel class of anti-tumor agents, immune checkpoint inhibitors (ICIs) targeting the interaction between programmed cell death 1 (PD-1) and programmed cell death ligand 1 (PD-L1) represent a major paradigm shift in cancer treatment. The use of ICIs such as PD-1 and PD-L1 inhibitors has been approved by Food and Drug Administration (FDA) and the National Medical Products Administration (NMPA) of China for treating many solid tumor types including melanoma, non-small cell lung cancer, small cell lung cancer, bladder cancer, and colorectal cancer (1). Although these drugs have shown impressive anti-tumor efficacy, they may be associated with a wide range of mild to severe or life-threatening characteristic adverse effects, termed autoimmune immune-related adverse events (irAEs). The most common irAEs occur in the skin, endocrine system, pulmonary, and gastrointestinal tract. The irAEs affecting the musculoskeletal system, cardiovascular system, and nervous system are relatively rare (2, 3). Myopathy induced by ICIs represents a multifaceted entity with diverse clinical features of the neuromuscular system, including myositis, myocarditis, general myasthenia, myasthenia gravis (MG), rhabdomyolysis, and dermatomyositis (4). Overlapping myositis with myocarditis, myasthenia, and ocular myositis has been reported in some literature (5, 6). However, only a few cases involved immune-mediated direct or indirect damage in the diaphragm (7). Here, we reported a rare case with extrahepatic cholangiocarcinoma who developed esophageal hiatal hernia resulting from clinically confirmed myositis accompanied by myocarditis and myasthenia after discontinuation of immunotherapy and steroid treatment, supporting the evidence of more generalized muscle involvement underlying clinical presentations. The severity for patients with myositis and myocarditis accompanied without MG is similar to those with MG. Physicians should therefore pay more attention to immunotherapy-induced myocarditis with myositis/MG overlap syndrome. Since we hypothesize diaphragmatic hiatal hernia as a potential consequence of immunotherapy-induced myositis, reports on hiatal hernias subsequent to immunotherapy-induced myositis are needed.

## Case description

Ethical approval: The study was approved by the First Affiliated Hospital of Shandong First Medical University (No: [2022]-S479). Written informed consent was obtained from the patient. Written informed consent was obtained from the participant for the publication of this case report (including all data and images).

**FIGURE 1:**
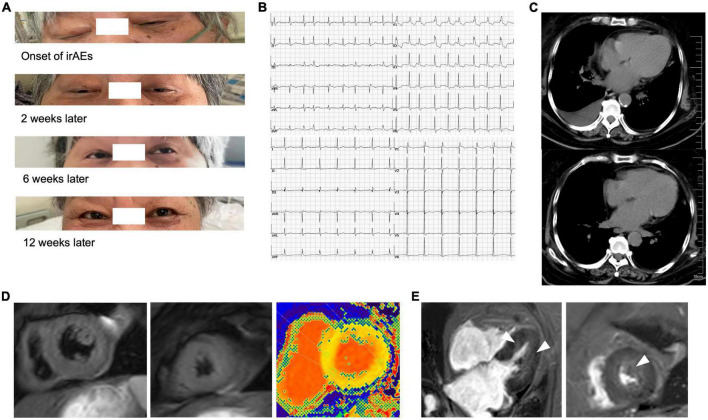
Eyelid ptosis and radiographical presentations of immune-related myocarditis. **(A)** Both eyelid ptosis occurred at the time of immune-related adverse events (irAEs) onset and improved gradually after the steroid treatment and intravenous immunoglobulin (IVIG). **(B)** The electrocardiogram showed ectopic heart rhythm and atrial arrhythmia before (upper) and after (lower) the steroid treatment. **(C)** Temporary right pleural infusion before (upper) and after (lower) the steroid treatment by CT scan. **(D)** In the third month after the onset of irAEs, the patient received a supplementary cardiac magnetic resonance (MR) examination revealing no edema and hyperemia (left and central), and quantitative myocardial T1 mapping was normal (right). **(E)** Delayed myocardial enhancement showed patchy enhancement that may be old myocardial injuries resulting from immune-related cardiac toxicity (arrow).

**FIGURE 2:**
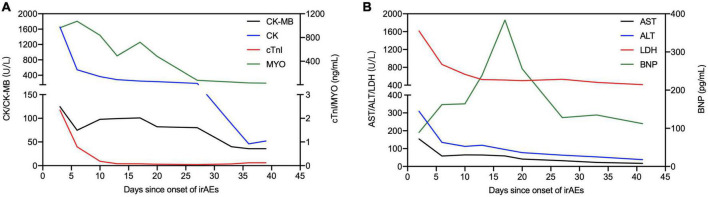
Illustration of main laboratory markers and steroid treatment following the onset of irAEs. **(A)** Dynamics of cardiac markers [creatine kinase (CK), creatine kinase isoenzyme (CK-MB), cardiac troponin I (cTnI), and myoglobin (MYO)]. **(B)** Dynamics of other biochemical markers [alanine aminotransferase (ALT), aspartate aminotransferase (AST), lactate dehydrogenase (LDH), and brain natriuretic peptide (BNP)].

**FIGURE 3:**
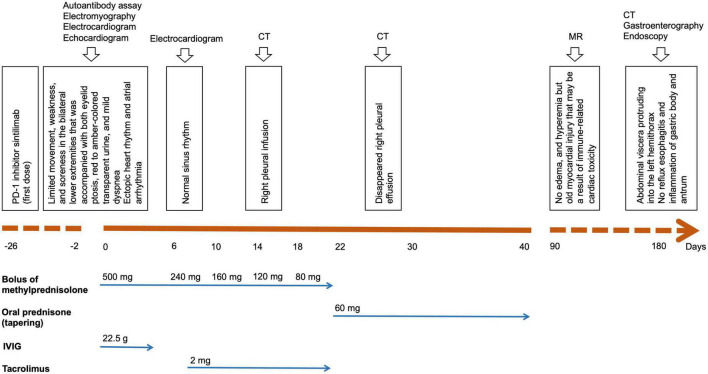
Patient timeline chart along with key dates or durations for clinical manifestations, investigations, and treatments with steroids and immunosuppressive agents.

In August 2020, a 71-year-old Chinese woman was initially diagnosed with unresectable extrahepatic cholangiocarcinoma and biliary obstruction. She received an endoscopically implantable stent and six cycles of first-line chemotherapy with gemcitabine and cisplatin with tolerated chemotherapy-related adverse effects from September 2020 to April 2021. Unfortunately, in April 2021, she switched to immunotherapy with PD-1 inhibitor sintilimab due to disease progression. A week later after immunotherapy, the patient developed fatigue and low back pain without any treatment. In May 2021, she began to suffer from limited movement, weakness, and soreness in the bilateral lower extremities that was accompanied with both eyelid ptosis, red to amber-colored transparent urine, and mild dyspnea (Figure 1A). This patient had no prior history of muscle weakness and neuromuscular disease with a normal body mass index of 20.2 kg/m^2^. No abnormality was found in electromyography, and only multiple ischemic infarcts were found in brain magnetic resonance (MR). The electrocardiogram showed ectopic heart rhythm and atrial arrhythmia (Figure 1B). She had a right pleural infusion (Figure 1C). The echocardiogram showed an left ventricular ejection fraction (LVEF) of 65% with normal left ventricular systolic and diastolic functions. Subsequent screening for MG antibodies showed negative ryanodine receptor antibody (RyR-Ab) and weakly positive acetylcholine receptor antibody (AChR-Ab, 0.93 nmol/l, normal range <0.45 nmol/l). All myositis-specific and myositis-related antibody profiling revealed negative antibodies. Laboratory examinations showed that creatine kinase (CK) was increased to 1,658 U/L (normal range <140 U/L), creatine kinase isoenzyme (CK-MB) to 124.49 U/L (normal range <25 U/L), cardiac troponin I (cTnI) to 2.35 ng/mL (normal range <0.034 ng/mL), and myoglobin (MYO) to 965.4 ng/mL (normal range <61.5 ng/mL) (Figure 2A). She also had an elevated alanine aminotransferase (ALT) level of 309.1 U/L (normal range <40 U/L), aspartate aminotransferase (AST) level of 154.9 U/L (normal range <35 U/L), and lactate dehydrogenase (LDH) level of 1,616 U/L (normal range <214 U/L), but her brain natriuretic peptide (BNP) was normal (Figure 2B). At that time, she failed to conduct the cardiac MR examination and endomyocardial biopsy due to her poor performance status and discomfort symptoms. She also refused to do further muscle biopsy at that time. Based on the clinical manifestations and laboratory test results, a clinical diagnosis of grade 4 ICI-induced myopathy, including myositis, myalgia, myasthenia, and myocarditis due to multiple muscle injuries in cardiac, skeletal, and ocular muscle, was made. However, immune-related MG was ruled out. She was subsequently initiated with pulse methylprednisolone at 500 mg/d and for 5 days with gradually decreasing doses and intravenous immunoglobulin (IVIG) at a dose of 0.4 g/kg/d for 5 days. A week later, she still complained of bilateral eyelid ptosis, but weakness and soreness in the bilateral lower extremities were significantly improved. At that time, tacrolimus at 3 mg/d was added. After 2 weeks of pulse methylprednisolone treatment, all her clinical symptoms associated with immune-related myopathy improved significantly with gradually declining biochemical biomarker levels and she was discharged. A computed tomography (CT) scan showed a temporary right pleural effusion and a slight elevation of BNP (Figures 1C, 2B). She was continuedly administrated with oral prednisolone at 60 mg/d for 4 weeks with a slow tapering for an additional 4 weeks (Figure 3). Because the patient’s performance status improves significantly, 3 months after the onset of immune-related irAEs, she received a supplementary cardiac MR examination revealing no edema and hyperemia, and quantitative myocardial T1 mapping was normal (Figure 1D). Delayed myocardial enhancement showed patchy enhancement that may be old myocardial injuries resulting from immune-related cardiac toxicity (Figure 1E).

**FIGURE 4:**
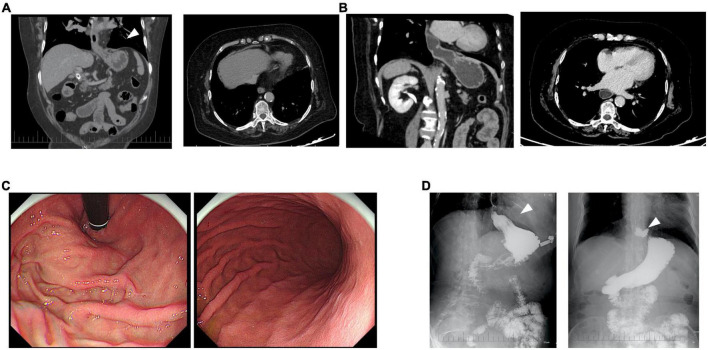
Clinical diagnosis of immune checkpoint inhibitor (ICI)-related esophageal hiatal hernia. At the sixth **(A)** and eighth month **(B)** after the onset of immune-related irAEs, a computed tomography (CT) scan for the first time showed abdominal viscera protruding into the left hemithorax (arrow). Upper gastroenterography and endoscopy showed no esophagitis and inflammation of the gastric body and antrum **(C)**, but a clinically confirmed diagnosis of sliding hiatal hernia of the esophagus (type I) was made (arrow) **(D)**.

In the third month following the PD-1 inhibitor treatment, all symptoms associated with immune-related myopathy disappeared, including weakness in the bilateral lower extremities, soreness, and bilateral eyelid ptosis, and all biochemical biomarkers were normal. CT scan showed her right pleural effusion improved completely (Figure 1C). She discontinued immunosuppressive agents and oral prednisolone and was treated with systemic chemotherapy in combination with an anti-angiogenic agent but without immunotherapy. In January 2022, the patient complained of repeated upper abdominal distension for several days. CT scan for the first time showed abdominal viscera protruding into the left hemithorax (Figures 4A,B), and an esophageal hiatal hernia was suspected. Further upper gastroenterography and endoscopy showed no esophagitis and inflammation of the gastric body and antrum (Figure 4C), but a clinically confirmed diagnosis of sliding hiatal hernia of the esophagus (type I) was made (Figure 4D). A gastrointestinal motility examination confirmed a diagnosis of gastroesophageal reflux disease. Because this patient had only mild symptoms associated with gastroesophageal reflux, she was suggested close monitoring with acid secretion blockade rather than immediate surgical intervention.

## Discussion

Immune checkpoint inhibitor-induced myopathy is characterized as a multifaceted entity with diverse clinical features of the neuromuscular system including myositis, myocarditis, myasthenic crisis, rhabdomyolysis, and dermatomyositis (4). Symptoms related to ICI-induced myopathy range from mild to severe toxicities, but the diagnosis of immune-related myopathy is often challenging because they may manifest as common symptoms such as cancer or other concurrent anti-tumor agent-related fatigue and weakness (4). The incidence of immune-related myocarditis is less than 1%, and most cases happen after initial one to two doses of ICIs and rapidly deteriorate, although some are asymptomatic with the elevation of cardiac marker levels alone. The incidence and mortality of myocarditis with cytotoxic T lymphocyte-associated antigen-4 (CTLA-4) inhibitor in combination with PD-1/PD-L1 inhibitor is higher than those with PD-1/PD-L1 inhibitor alone (8). A recent review article demonstrates that MG is the most common ICI-related neuromuscular adverse effect (26.8%), followed by myositis (25.6%), and Guillain–Barre syndrome (18.3%) (9). ICI-induced myositis often has a broad spectrum, ranging from mild symptoms to severe or life-threatening complications (10). Based on the pharmacovigilance database of the World Health Organization, Allenbach et al. (10) identified 465 cases of myositis with a total incidence rate of <1%. Among rheumatic irAEs (arthritis, myositis, sarcoidosis, Sjogren’s syndrome, scleroderma, and rheumatic polymyalgia), myositis has the shortest median onset time (median 31 days; range 19.2–57.8) and the highest case fatality rate (24%), especially when it is associated with myocarditis (57%) (10). In Pathak et al.’s (11) study, most cases developed myositis symptoms after one ICI treatment. Compared with primary multiple autoimmune myositis (12), most cases progress rapidly. Elevated CK levels were reported in all cases. Based on VigiBase analysis (13), in 180 patients with myositis associated with ICI treatment, the mortality rate was significantly higher than that of patients with idiopathic autoimmune myopathy (21.2 vs. <10%). Serious complications (defined as long hospital stays, life-threatening events, or residual disability) occurred in 49.4% of patients.

Furthermore, some reported cases presented with simultaneous myocarditis, myositis, and MG secondary to ICI which may represent an overlap syndrome. A report involving 101 cases of ICI-related myocarditis showed that the most commonly occurring concurrent irAEs were myositis (25%) and MG (10%) (5). Patients with ICI-induced myositis frequently complain of oculomotor weakness or eyelid ptosis because of ocular involvement, which can confound the diagnosis of ICI-induced MG (6). Therefore, the complexity and severity of these overlapping neuromuscular toxicities highlight the urgent need for clinicians to suspect and diagnose multiple simultaneous irAEs and conduct further multidisciplinary approach. Previously reported studies showed that 24.7% (39/158) of cases with ICI-induced myositis and myocarditis developed simultaneous MG. The mortality for patients who had myositis and myocarditis accompanied with or without MG was 32.8 and 25.6%, respectively (Supplementary Table 1). As a result, the severity for patients with myositis and myocarditis accompanied without MG is similar to those with MG. After all, myocarditis had the highest fatality rate of 39.7% in 131 reported cases regardless of the co-occurrence of MG (14).

Monitoring the levels of cardiac Troponin T (cTnT) or cardiac Troponin I (cTnI) is helpful to differentiate cardiac from skeletal damage induced by ICIs. Muscle-specific antibody profiling can be used to differentiate baseline autoimmune disease. Based on the clinical presentations and laboratory findings, our patient represents a diagnosis of simultaneous myositis and myocarditis. This patient had increased levels of cTnI, CK, MYO, and elevated liver enzymes as the result of simultaneous cardiac and skeletal muscle damage. ICI-induced myopathy has been described to be associated with CD8^+^ T lymphocyte and macrophage infiltration (some like granulomas) and necrotizing myositis (4). Matas et al. (15) reported obvious necrosis, macrophages, and muscle regeneration with perivascular inflammatory infiltrates with a large component of macrophagic cells in the pathological review of muscle biopsies of nine patients. Myocarditis induced by ICI has similar characteristics to T cells (especially CD8^+^ T cells). Unfortunately, pathological analysis of myocarditis or myositis was not available because this patient refused to perform muscle biopsy and endomyocardial biopsy, but a subsequent cardiac MR examination revealed an old myocardial injury that may be a result of immune-related cardiac toxicity.

Diaphragmatic dysfunction as immune-mediated toxicity of ICI therapy is a rarely reported side effect. The presenting symptom of diaphragmatic dysfunction is often dyspnea or orthopnea. At Mayo Clinic, three patients with diaphragmatic dysfunction in the setting of ICI therapy were successfully treated without mortality (16). One patient had a significant, systemic inflammatory response after one cycle of a combination of ipilimumab and nivolumab therapy with a diffuse myopathic process involving the diaphragm and likely the myocardium. It was not an isolated irAE, but rather, was part of a systemic inflammatory process with multiple organ involvement. In the eighth month following the PD-1 inhibitor treatment, the present patient developed a hiatal hernia. Esophageal hiatal hernia refers to the hernia formed by any abdominal tissue structure except the esophagus entering the thoracic cavity through the enlarged esophageal hiatus (17). Age and increased body mass index are characterized as key risk factors for hiatal hernia. The enlargement of the diaphragm-esophageal hiatus is due to the gradual loss of elastin, which makes the ligament around the esophagus and diaphragm relax, resulting in the enlargement of esophageal hiatus and the formation of hiatal hernia. Here, we considered that impaired diaphragm function as a cause of the hiatal hernia was only a clinical diagnosis. First, a hiatal hernia is a relatively frequent clinical disorder, which may occur spontaneously, certainly in a woman with an age of older than 50 years, but this patient had a normal body mass index of 20.2 kg/m^2^. Second, she did not have a history of esophageal hiatal hernia and damage of the diaphragm based on her baseline CT scan. Third, ICI-induced myositis can involve multiple striated muscles including cardiac muscle, skeletal muscles, and ocular muscles, as well as the diaphragm, leading to the enlargement of diaphragm-esophageal hiatus and subsequent formation of esophageal hiatal hernia. Recently, Tajima et al. (7) reported a case of fatal fulminant inflammation in the diaphragm resulting from pembrolizumab-related myopathy. Histological analysis showed that massive infiltration of inflammatory cells and muscle fiber necrosis occurred in the diaphragm of the patient, supporting a possible link between ICI-related elated myopathy and diaphragm damage. Thus, the weakness and damage of the diaphragm resulting from immunotherapy-related myopathy or myositis may lead to esophageal hiatal hernia in the present patient. However, here we did not provide substantiation for the causal link with the myositis. Future pathological investigations of the diaphragm are helpful to prove this causal link.

Mild to intermediate irAEs (grade 1 to 2) are clinically well manageable, but more severe cases (grades 3 to 4) are usually life-threatening and require high-dose steroids followed by slow tapering within a few weeks. In cases with severe or steroid-refractory irAEs, most guidelines recommended the administration of immunomodulatory agents such as mycophenolate mofetil, tacrolimus, and tumor necrosis factor-α (TNF-α) inhibitors, as well as anti-thymocyte globulin (18). A multicenter case series by Moreira et al. (19) showed that in 20 cases of myositis (including cases with overlapping MG, polyneuropathy, or myocarditis), 15 patients (79%) were treated with high-dose steroids, and 50% had a complete remission of clinical symptoms (19). In addition to steroids, most patients with myocarditis and MG received IVIG and plasma exchange. In our case study, this patient was initially treated with pulse methylprednisolone and IVIG, and oral tacrolimus was added to steroid therapy when she still suffered from bilateral eyelid ptosis. She received the treatment with steroids and an immunomodulatory agent for a total of 10 weeks. She also restarted systemic chemotherapy in combination with an anti-angiogenic agent due to the significant improvement in clinical presentations. In terms of treatment for esophageal hiatal hernia, different treatment methods should be selected according to patient’s condition and classification. This patient with a sliding hiatal hernia had mild symptoms and should be treated conservatively, and continuing steroids and the immunomodulatory agent is unnecessary. The sliding hiatal hernia seemed to be an anatomical change of the upper gastrointestinal tract and diaphragm as a rare and delayed immune-related neuromuscular manifestation. Laparoscopic surgery should be considered if she experiences gastroesophageal reflux disease symptoms in future. Fortunately, this patient’s esophageal hiatal hernia did not progress. She was suggested close monitoring with acid secretion blockade, and her cancer is well controlled.

## Conclusion

Here, we reported a case of concurrent myocarditis, myositis, diaphragmatic dysfunction, and esophageal hiatal hernia following treatment with PD-1 inhibitor sintilimab. Autoimmune myocarditis and neuromuscular side effects induced by ICI although rare can be severe and sometimes fatal. The severity for patients with myositis and myocarditis accompanied without MG is similar to those with MG. Early diagnosis and prompt treatment of myocarditis and myositis/MG overlap syndrome can decrease morbidity and possibly mortality because patients often respond well to corticosteroid therapy and other aggressive immunosuppressive agents. Considering the use of PD-1 inhibitors is increasing in cancer patients, physicians should therefore pay more attention to immunotherapy-induced myocarditis with myositis/MG overlap syndrome. Since we hypothesize diaphragmatic hiatal hernia as a potential consequence of immunotherapy-induced myositis, reports on hiatal hernias subsequent to immunotherapy-induced myositis are needed. Future pathological investigations of the diaphragm are helpful to prove this causal link with myositis. Further studies to characterize risk factors and management strategies are also urgently needed.

## Data availability statement

The original contributions presented in this study are included in the article/Supplementary material, further inquiries can be directed to the corresponding author.

## Ethics statement

The studies involving human participants were reviewed and approved by the First Affiliated Hospital of Shandong First Medical University (No: [2022]-S479). The patients/participants provided their written informed consent to participate in this study. Written informed consent was obtained from the individual(s) for the publication of any potentially identifiable images or data included in this article.

## Author contributions

BY wrote the manuscript and prepared the figures. JX, XW, XL, YG, JC, and KL collected the clinical data. PH performed the radiographical examinations. JW conceived and wrote the manuscript. All authors contributed in their order in writing the manuscript, read, and approved the final manuscript.
