# Supplementary material for: Myocarditis and myositis/myasthenia gravis overlap syndrome induced by immune checkpoint inhibitor followed by esophageal hiatal hernia: A case report and review of the literature
Source: Front Med (Lausanne). 2022 Nov 15;9:950801. doi: 10.3389/fmed.2022.950801 (PMC9705572; doi:10.3389/fmed.2022.950801)
Supplement: Supplementary file 2 [file Table_2.docx]

Ang, E., Mweempwa, A., Heron, C., Ahn, Y., Rivalland, G., Ha, L.Y., et al. (2021). Cardiac Troponin I and T in Checkpoint Inhibitor-associated Myositis and Myocarditis. *J Immunother* 44: 162-163. doi: 10.1097/cji.0000000000000356

Arangalage, D., Delyon, J., Lermuzeaux, M., Ekpe, K., Ederhy, S., Pages, C., et al. (2017). Survival After Fulminant Myocarditis Induced by Immune-Checkpoint Inhibitors. *Ann Intern Med* 167: 683-684. doi: 10.7326/l17-0396

Arora, P., Talamo, L., Dillon, P., Gentzler, R.D., Millard, T., Salerno, M., et al. (2020). Severe combined cardiac and neuromuscular toxicity from immune checkpoint blockade: an institutional case series. *Cardiooncology* 6: 21. doi: 10.1186/s40959-020-00076-6

Bai, J., Li, D., Yang, P., Xu, K., Wang, Y., Li, Q., et al. (2021). Camrelizumab-Related Myocarditis and Myositis With Myasthenia Gravis: A Case Report and Literature Review. *Front Oncol* 11: 778185. doi: 10.3389/fonc.2021.778185

Bawek, S.J., Ton, R., Mcgovern-Poore, M., Khoncarly, B., and Narvel, R. (2021). Nivolumab-Induced Myasthenia Gravis Concomitant With Myocarditis, Myositis, and Hepatitis. *Cureus* 13: e18040. doi: 10.7759/cureus.18040

Behling, J., Kaes, J., Münzel, T., Grabbe, S., and Loquai, C. (2017). New-onset third-degree atrioventricular block because of autoimmune-induced myositis under treatment with anti-programmed cell death-1 (nivolumab) for metastatic melanoma. *Melanoma Res* 27: 155-158. doi: 10.1097/cmr.0000000000000314

Bhatlapenumarthi, V., Patwari, A., and Harb, A.J. (2021). Immune-related adverse events and immune checkpoint inhibitor tolerance on rechallenge in patients with irAEs: a single-center experience. *J Cancer Res Clin Oncol* 147: 2789-2800. doi: 10.1007/s00432-021-03610-w

Boisseau, W., Touat, M., Berzero, G., Savatovsky, J., Marabelle, A., Touitou, V., et al. (2017). Safety of treatment with nivolumab after ipilimumab-related meningoradiculitis and bilateral optic neuropathy. *Eur J Cancer* 83: 28-31. doi: 10.1016/j.ejca.2017.05.036

Bukamur, H.S., Mezughi, H., Karem, E., Shahoub, I., and Shweihat, Y. (2019). Nivolumab-induced Third Degree Atrioventricular Block in a Patient with Stage IV Squamous Cell Lung Carcinoma. *Cureus* 11: e4869. doi: 10.7759/cureus.4869

Cao, J., Li, Q., Zhi, X., Yang, F., Zhu, W., Zhou, T., et al. (2021). Pembrolizumab-induced autoimmune Stevens-Johnson syndrome/toxic epidermal necrolysis with myositis and myocarditis in a patient with esophagogastric junction carcinoma: a case report. *Transl Cancer Res* 10: 3870-3876. doi: 10.21037/tcr-21-470

Carausu, M., Beddok, A., Langer, A., Girard, N., Bidard, F.C., Massiani, M.A., et al. (2019). Radiation myelitis after pembrolizumab administration, with favorable clinical evolution and safe rechallenge: a case report and review of the literature. *J Immunother Cancer* 7: 317. doi: 10.1186/s40425-019-0803-x

Cham, J., Ng, D., and Nicholson, L. (2021). Durvalumab-induced myocarditis, myositis, and myasthenia gravis: a case report. *J Med Case Rep* 15: 278. doi: 10.1186/s13256-021-02858-7

Charles, J., Giovannini, D., Terzi, N., Schwebel, C., Sturm, N., Masson, D., et al. (2019). Multi-organ failure induced by Nivolumab in the context of allo-stem cell transplantation. *Exp Hematol Oncol* 8: 8. doi: 10.1186/s40164-019-0132-2

Chen, Q., Huang, D.S., Zhang, L.W., Li, Y.Q., Wang, H.W., and Liu, H.B. (2018). Fatal myocarditis and rhabdomyolysis induced by nivolumab during the treatment of type B3 thymoma. *Clin Toxicol (Phila)* 56: 667-671. doi: 10.1080/15563650.2017.1401079

Chen, Y., Jia, Y., Liu, Q., Shen, Y., Zhu, H., Dong, X., et al. (2021). Myocarditis related to immune checkpoint inhibitors treatment: two case reports and literature review. *Ann Palliat Med* 10: 8512-8517. doi: 10.21037/apm-20-2620

Cuzzubbo, S., Tetu, P., Guegan, S., Ursu, R., Belin, C., Sirven Villaros, L., et al. (2020). Reintroduction of immune-checkpoint inhibitors after immune-related meningitis: a case series of melanoma patients. *J Immunother Cancer* 8: e001034. doi: 10.1136/jitc-2020-001034

Delyon, J., Brunet-Possenti, F., Leonard-Louis, S., Arangalage, D., Baudet, M., Baroudjian, B., et al. (2019). Immune checkpoint inhibitor rechallenge in patients with immune-related myositis. *Ann Rheum Dis* 78: e129. doi: 10.1136/annrheumdis-2018-214336

Dubey, D., David, W.S., Amato, A.A., Reynolds, K.L., Clement, N.F., Chute, D.F., et al. (2019). Varied phenotypes and management of immune checkpoint inhibitor-associated neuropathies. *Neurology* 93: e1093-e1103. doi: 10.1212/wnl.0000000000008091

Fazal, M., Prentice, D.A., Kho, L.K., and Fysh, E. (2020). Nivolumab-associated myositis myocarditis and myasthenia and anti-striated muscle antibodies. *Intern Med J* 50: 1003-1006. doi: 10.1111/imj.14946

Fazel, M., and Jedlowski, P.M. (2019). Severe Myositis, Myocarditis, and Myasthenia Gravis with Elevated Anti-Striated Muscle Antibody following Single Dose of Ipilimumab-Nivolumab Therapy in a Patient with Metastatic Melanoma. *Case Reports Immunol* 2019: 2539493. doi: 10.1155/2019/2539493

Fellner, A., Makranz, C., Lotem, M., Bokstein, F., Taliansky, A., Rosenberg, S., et al. (2018). Neurologic complications of immune checkpoint inhibitors. *J Neurooncol* 137: 601-609. doi: 10.1007/s11060-018-2752-5

Fuentes-Antrás, J., Peinado, P., Guevara-Hoyer, K., Del Arco, C.D., Sánchez-Ramón, S., and Aguado, C. (2020). Fatal autoimmune storm after a single cycle of anti-PD-1 therapy: A case of lethal toxicity but pathological complete response in metastatic lung adenocarcinoma. *Hematol Oncol Stem Cell Ther* 30098-4: 30098-30094. doi: 10.1016/j.hemonc.2020.04.006

Fukasawa, Y., Sasaki, K., Natsume, M., Nakashima, M., Ota, S., Watanabe, K., et al. (2017). Nivolumab-Induced Myocarditis Concomitant with Myasthenia Gravis. *Case Rep Oncol* 10: 809-812. doi: 10.1159/000479958

Galmiche, S., Lheure, C., Kramkimel, N., Franck, N., Boitier, F., Dupin, N., et al. (2019). Encephalitis induced by immune checkpoint inhibitors in metastatic melanoma: a monocentric retrospective study. *J Eur Acad Dermatol Venereol* 33: e440-e443. doi: 10.1111/jdv.15756

Gerdes, L.A., Held, K., Beltrán, E., Berking, C., Prinz, J.C., Junker, A., et al. (2016). CTLA4 as Immunological Checkpoint in the Development of Multiple Sclerosis. *Ann Neurol* 80: 294-300. doi: 10.1002/ana.24715

González-Ferrero, T., Vargas-Osorio, K., and González-Juanatey, J.R. (2022). Fulminant myocarditis with myositis after treatment with immune checkpoint inhibitors. *Med Clin (Barc)* 158: 140-141. doi: 10.1016/j.medcli.2021.04.014

Gravbrot, N., Scherer, K., and Sundararajan, S. (2019). Safe Transition to Pembrolizumab following Ipilimumab-Induced Guillain-Barré Syndrome: A Case Report and Review of the Literature. *Case Rep Oncol Med* 2019: 5490707. doi: 10.1155/2019/5490707

Hellman, J.B., Traynis, I., and Lin, L.K. (2019). Pembrolizumab and epacadostat induced fatal myocarditis and myositis presenting as a case of ptosis and ophthalmoplegia. *Orbit* 38: 244-247. doi: 10.1080/01676830.2018.1490439

Hoa, S., Laaouad, L., Roberts, J., Ennis, D., Ye, C., Al Jumaily, K., et al. (2021). Preexisting autoimmune disease and immune-related adverse events associated with anti-PD-1 cancer immunotherapy: a national case series from the Canadian Research Group of Rheumatology in Immuno-Oncology. *Cancer Immunol Immunother* 70: 2197-2207. doi: 10.1007/s00262-021-02851-5

Hu, X., Wei, Y., and Shuai, X. (2021). Case Report: Glucocorticoid Effect Observation in a Ureteral Urothelial Cancer Patient With ICI-Associated Myocarditis and Multiple Organ Injuries. *Front Immunol* 12: 799077. doi: 10.3389/fimmu.2021.799077

Imai, R., Ono, M., Nishimura, N., Suzuki, K., Komiyama, N., and Tamura, T. (2019). Fulminant Myocarditis Caused by an Immune Checkpoint Inhibitor: A Case Report With Pathologic Findings. *J Thorac Oncol* 14: e36-e38. doi: 10.1016/j.jtho.2018.10.156

Jespersen, M.S., Fanø, S., Stenør, C., and Møller, A.K. (2021). A case report of immune checkpoint inhibitor-related steroid-refractory myocarditis and myasthenia gravis-like myositis treated with abatacept and mycophenolate mofetil. *Eur Heart J Case Rep* 5: ytab342. doi: 10.1093/ehjcr/ytab342

Jeyakumar, N., Etchegaray, M., Henry, J., Lelenwa, L., Zhao, B., Segura, A., et al. (2020). The Terrible Triad of Checkpoint Inhibition: A Case Report of Myasthenia Gravis, Myocarditis, and Myositis Induced by Cemiplimab in a Patient with Metastatic Cutaneous Squamous Cell Carcinoma. *Case Reports Immunol* 2020: 5126717. doi: 10.1155/2020/5126717

Johnson, D.B., Balko, J.M., Compton, M.L., Chalkias, S., Gorham, J., Xu, Y., et al. (2016). Fulminant Myocarditis with Combination Immune Checkpoint Blockade. *The New England journal of medicine* 375: 1749-1755. doi: 10.1056/NEJMoa1609214

Kadota, H., Gono, T., Shirai, Y., Okazaki, Y., Takeno, M., and Kuwana, M. (2019). Immune Checkpoint Inhibitor-Induced Myositis: a Case Report and Literature Review. *Curr Rheumatol Rep* 21: 10. doi: 10.1007/s11926-019-0811-3

Konstantina, T., Konstantinos, R., Anastasios, K., Anastasia, M., Eleni, L., Ioannis, S., et al. (2019). Fatal adverse events in two thymoma patients treated with anti-PD-1 immune check point inhibitor and literature review. *Lung Cancer* 135: 29-32. doi: 10.1016/j.lungcan.2019.06.015

Larkin, J., Chmielowski, B., Lao, C.D., Hodi, F.S., Sharfman, W., Weber, J., et al. (2017). Neurologic Serious Adverse Events Associated with Nivolumab Plus Ipilimumab or Nivolumab Alone in Advanced Melanoma, Including a Case Series of Encephalitis. *Oncologist* 22: 709-718. doi: 10.1634/theoncologist.2016-0487

Liang, S., Yang, J., Lin, Y., Li, T., Zhao, W., Zhao, J., et al. (2021). Immune Myocarditis Overlapping With Myasthenia Gravis Due to Anti-PD-1 Treatment for a Chordoma Patient: A Case Report and Literature Review. *Front Immunol* 12: 682262. doi: 10.3389/fimmu.2021.682262

Lie, G., Weickhardt, A., Kearney, L., Lam, Q., John, T., Liew, D., et al. (2020). Nivolumab resulting in persistently elevated troponin levels despite clinical remission of myocarditis and myositis in a patient with malignant pleural mesothelioma: case report. *Transl Lung Cancer Res* 9: 360-365. doi: 10.21037/tlcr.2020.02.05

Lipe, D.N., Galvis-Carvajal, E., Rajha, E., Wechsler, A.H., and Gaeta, S. (2021). Immune checkpoint inhibitor-associated myasthenia gravis, myositis, and myocarditis overlap syndrome. *Am J Emerg Med* 46: 51-55. doi: 10.1016/j.ajem.2021.03.005

Liu, W.K., Naban, N., Kaul, A., Patel, N., and Fusi, A. (2021). Life-threatening polymyositis with spontaneous hematoma induced by nivolumab in a patient with previously resected melanoma. *Melanoma Res* 31: 85-87. doi: 10.1097/cmr.0000000000000706

Luo, Y.B., Tang, W., Zeng, Q., Duan, W., Li, S., Yang, X., et al. (2021). Case Report: The Neuromusclar Triad of Immune Checkpoint Inhibitors: A Case Report of Myositis, Myocarditis, and Myasthenia Gravis Overlap Following Toripalimab Treatment. *Front Cardiovasc Med* 8: 714460. doi: 10.3389/fcvm.2021.714460

Martinez-Calle, N., Rodriguez-Otero, P., Villar, S., Mejías, L., Melero, I., Prosper, F., et al. (2018). Anti-PD1 associated fulminant myocarditis after a single pembrolizumab dose: the role of occult pre-existing autoimmunity. *Haematologica* 103: e318-e321. doi: 10.3324/haematol.2017.185777

Matsui, H., Kawai, T., Sato, Y., Ishida, J., Kadowaki, H., Akiyama, Y., et al. (2020). A Fatal Case of Myocarditis Following Myositis Induced by Pembrolizumab Treatment for Metastatic Upper Urinary Tract Urothelial Carcinoma. *Int Heart J* 61: 1070-1074. doi: 10.1536/ihj.20-162

Mehta, J.J., Maloney, E., Srinivasan, S., Seitz, P., and Cannon, M. (2017). Myasthenia Gravis Induced by Nivolumab: A Case Report. *Cureus* 9: e1702. doi: 10.7759/cureus.1702

Monge, C., Maeng, H., Brofferio, A., Apolo, A.B., Sathya, B., Arai, A.E., et al. (2018). Myocarditis in a patient treated with Nivolumab and PROSTVAC: a case report. *J Immunother Cancer* 6: 150. doi: 10.1186/s40425-018-0473-0

Muralikrishnan, S., Ronan, L.K., Coker, S., Rauschkolb, P.K., and Shirai, K. (2020). Treatment Considerations for Patients with Unresectable Metastatic Melanoma Who Develop Pembrolizumab-Induced Guillain-Barré Toxicity: A Case Report. *Case Rep Oncol* 13: 43-48. doi: 10.1159/000504930

Nakagomi, Y., Tajiri, K., Shimada, S., Li, S., Inoue, K., Murakata, Y., et al. (2022). Immune Checkpoint Inhibitor-Related Myositis Overlapping With Myocarditis: An Institutional Case Series and a Systematic Review of Literature. *Front Pharmacol* 13: 884776. doi: 10.3389/fphar.2022.884776

Nakatani, Y., Tanaka, N., Enami, T., Minami, S., Okazaki, T., and Komuta, K. (2018). Lambert-Eaton Myasthenic Syndrome Caused by Nivolumab in a Patient with Squamous Cell Lung Cancer. *Case Rep Neurol* 10: 346-352. doi: 10.1159/000494078

Nasr, F., El Rassy, E., Maalouf, G., Azar, C., Haddad, F., Helou, J., et al. (2018). Severe ophthalmoplegia and myocarditis following the administration of pembrolizumab. *Eur J Cancer* 91: 171-173. doi: 10.1016/j.ejca.2017.11.026

Ono, R., Iwai, Y., Yamazaki, T., Takahashi, H., Hori, Y., Fukushima, K., et al. (2022). Nivolumab-induced Myositis and Myocarditis with Positive Anti-titin Antibody and Anti-voltage-gated Potassium Channel Kv1.4 Antibody: A Case Report. *Intern Med* 1: 1-7. doi: 10.2169/internalmedicine.8772-21

Plaçais, L., Michot, J.M., Champiat, S., Romano-Martin, P., Baldini, C., Joao, M.S., et al. (2021). Neurological complications induced by immune checkpoint inhibitors: a comprehensive descriptive case-series unravelling high risk of long-term sequelae. *Brain Commun* 3: fcab220. doi: 10.1093/braincomms/fcab220

Raskin, J., Masrori, P., Cant, A., Snoeckx, A., Hiddinga, B., Kohl, S., et al. (2017). Recurrent dysphasia due to nivolumab-induced encephalopathy with presence of Hu autoantibody. *Lung Cancer* 109: 74-77. doi: 10.1016/j.lungcan.2017.05.002

Rota, E., Varese, P., Agosti, S., Celli, L., Ghiglione, E., Pappalardo, I., et al. (2019). Concomitant myasthenia gravis, myositis, myocarditis and polyneuropathy, induced by immune-checkpoint inhibitors: A life-threatening continuum of neuromuscular and cardiac toxicity. *eNeurologicalSci* 14: 4-5. doi: 10.1016/j.ensci.2018.11.023

Saibil, S.D., Bonilla, L., Majeed, H., Sotov, V., Hogg, D., Chappell, M.A., et al. (2019). Fatal myocarditis and rhabdomyositis in a patient with stage IV melanoma treated with combined ipilimumab and nivolumab. *Curr Oncol* 26: e418-e421. doi: 10.3747/co.26.4381

Saishu, Y., Yoshida, T., Seino, Y., and Nomura, T. (2022). Nivolumab-related myasthenia gravis with myositis requiring prolonged mechanical ventilation: a case report. *J Med Case Rep* 16: 61. doi: 10.1186/s13256-022-03286-x

Sanchez-Sancho, P., Selva-O'callaghan, A., Trallero-Araguás, E., Ros, J., and Montoro, B. (2021). Myositis and myasteniform syndrome related to pembrolizumab. *BMJ Case Rep* 14: e241766. doi: 10.1136/bcr-2021-241766

Sechi, E., Markovic, S.N., Mckeon, A., Dubey, D., Liewluck, T., Lennon, V.A., et al. (2020). Neurologic autoimmunity and immune checkpoint inhibitors: Autoantibody profiles and outcomes. *Neurology* 95: e2442-e2452. doi: 10.1212/wnl.0000000000010632

Sessums, M., Yarrarapu, S., Guru, P.K., and Sanghavi, D.K. (2020). Atezolizumab-induced myositis and myocarditis in a patient with metastatic urothelial carcinoma. *BMJ Case Rep* 13: e236357. doi: 10.1136/bcr-2020-236357

Shah, M., Tayar, J.H., Abdel-Wahab, N., and Suarez-Almazor, M.E. (2019). Myositis as an adverse event of immune checkpoint blockade for cancer therapy. *Semin Arthritis Rheum* 48: 736-740. doi: 10.1016/j.semarthrit.2018.05.006

Shindo, A., Yamasaki, M., Uchino, K., and Yamasaki, M. (2022). Asymptomatic Myocarditis with Mild Cardiac Marker Elevation Following Nivolumab-Induced Myositis. *Int Heart J* 63: 180-183. doi: 10.1536/ihj.21-653

Shirai, T., Kiniwa, Y., Sato, R., Sano, T., Nakamura, K., Mikoshiba, Y., et al. (2019). Presence of antibodies to striated muscle and acetylcholine receptor in association with occurrence of myasthenia gravis with myositis and myocarditis in a patient with melanoma treated with an anti-programmed death 1 antibody. *Eur J Cancer* 106: 193-195. doi: 10.1016/j.ejca.2018.10.025

So, H., Ikeguchi, R., Kobayashi, M., Suzuki, M., Shimizu, Y., and Kitagawa, K. (2019). PD-1 inhibitor-associated severe myasthenia gravis with necrotizing myopathy and myocarditis. *J Neurol Sci* 399: 97-100. doi: 10.1016/j.jns.2019.02.023

Soror, N.N., Hemrock, L., Shah, P., Loges, R.J., and Tharu, B. (2021). Brain Stem Encephalitis in a Patient With Recurrent Small Cell Lung Cancer Treated With Immune Checkpoint Inhibitor: Case Presentation and Review of the Literature. *Cureus* 13: e13034. doi: 10.7759/cureus.13034

Spain, L., Walls, G., Messiou, C., Turajlic, S., Gore, M., and Larkin, J. (2017). Efficacy and toxicity of rechallenge with combination immune checkpoint blockade in metastatic melanoma: a case series. *Cancer Immunol Immunother* 66: 113-117. doi: 10.1007/s00262-016-1926-2

Swali, R. (2020). Pembrolizumab-induced Myositis in the Setting of Metastatic Melanoma: An Increasingly Common Phenomenon. *J Clin Aesthet Dermatol* 13: 44-45.

Szuchan, C., Elson, L., Alley, E., Leung, K., Camargo, A.L., Elimimian, E., et al. (2020). Checkpoint inhibitor-induced myocarditis and myasthenia gravis in a recurrent/metastatic thymic carcinoma patient: a case report. *Eur Heart J Case Rep* 4: 1-8. doi: 10.1093/ehjcr/ytaa051

Taliansky, A., Furman, O., Gadot, M., Urban, D., Bar, J., Shapira-Frumer, R., et al. (2021). Immune checkpoint inhibitors-related encephalitis in melanoma and non-melanoma cancer patients: a single center experience. *Support Care Cancer* 29: 7563-7568. doi: 10.1007/s00520-021-06331-5

Tedbirt, B., De Pontville, M., Branger, P., Picard, C., Baroudjian, B., Lebbé, C., et al. (2019). Rechallenge of immune checkpoint inhibitor after pembrolizumab-induced myasthenia gravis. *Eur J Cancer* 113: 72-74. doi: 10.1016/j.ejca.2019.03.006

Thouvenin, L., Olivier, T., Banna, G., Addeo, A., and Friedlaender, A. (2021). Immune checkpoint inhibitor-induced aseptic meningitis and encephalitis: a case-series and narrative review. *Ther Adv Drug Saf* 12: 20420986211004745. doi: 10.1177/20420986211004745

Todo, M., Kaneko, G., Shirotake, S., Shimada, Y., Nakano, S., Okabe, T., et al. (2020). Pembrolizumab-induced myasthenia gravis with myositis and presumable myocarditis in a patient with bladder cancer. *IJU Case Rep* 3: 17-20. doi: 10.1002/iju5.12128

Tomoaia, R., Beyer, R., Pop, D., Minciună, I.A., and Dădârlat-Pop, A. (2020). Fatal association of fulminant myocarditis and rhabdomyolysis after immune checkpoint blockade. *Eur J Cancer* 132: 224-227. doi: 10.1016/j.ejca.2020.03.003

Valenti-Azcarate, R., Esparragosa Vazquez, I., Toledano Illan, C., Idoate Gastearena, M.A., and Gállego Pérez-Larraya, J. (2020). Nivolumab and Ipilimumab-induced myositis and myocarditis mimicking a myasthenia gravis presentation. *Neuromuscul Disord* 30: 67-69. doi: 10.1016/j.nmd.2019.10.006

Veccia, A., Kinspergher, S., Grego, E., Peterlana, D., Berti, A., Tranquillini, E., et al. (2020). Myositis and myasthenia during nivolumab administration for advanced lung cancer: a case report and review of the literature. *Anticancer Drugs* 31: 540-544. doi: 10.1097/cad.0000000000000903

Velasco, R., Villagrán, M., Jové, M., Simó, M., Vilariño, N., Alemany, M., et al. (2021). Encephalitis Induced by Immune Checkpoint Inhibitors: A Systematic Review. *JAMA Neurol* 78: 864-873. doi: 10.1001/jamaneurol.2021.0249

Vogrig, A., Muñiz-Castrillo, S., Joubert, B., Picard, G., Rogemond, V., Marchal, C., et al. (2020). Central nervous system complications associated with immune checkpoint inhibitors. *J Neurol Neurosurg Psychiatry* 91: 772-778. doi: 10.1136/jnnp-2020-323055

Vogrig, A., Muñiz-Castrillo, S., Joubert, B., Picard, G., Rogemond, V., Skowron, F., et al. (2021). Cranial Nerve Disorders Associated With Immune Checkpoint Inhibitors. *Neurology* 96: e866-e875. doi: 10.1212/wnl.0000000000011340

Wai Siu, D.H., O'neill, R.S., Harris, C.A., Wang, J., Ardolino, L., Downton, T., et al. (2022). Immune checkpoint inhibitor-induced myocarditis, myositis, myasthenia gravis and transaminitis: a case series and review. *Immunotherapy* 14: 511-520. doi: 10.2217/imt-2021-0225

Weill, A., Delyon, J., Descamps, V., Deschamps, L., Dinulescu, M., Dupuy, A., et al. (2021). Treatment strategies and safety of rechallenge in the setting of immune checkpoint inhibitors-related myositis: a national multicentre study. *Rheumatology (Oxford)* 60: 5753-5764. doi: 10.1093/rheumatology/keab249

Witham, D., Knauss, S., Marek, A., Dreger, H., Harms, L., Joehrens, K., et al. (2017). Acute myocarditis and myositis after immune checkpoint inhibition. *Eur J Heart Fail* 19: 16.

Wu, N.C., Feng, Y.H., Kuo, Y.H., Chen, W.Y., Wu, H.C., Huang, C.T., et al. (2022). Clinical Features and Outcomes of Immune Checkpoint Inhibitor-Associated Cardiovascular Toxicities. *Acta Cardiol Sin* 38: 39-46. doi: 10.6515/acs.202201_38(1).20210830b

Xing, Q., Zhang, Z.W., Lin, Q.H., Shen, L.H., Wang, P.M., Zhang, S., et al. (2020). Myositis-myasthenia gravis overlap syndrome complicated with myasthenia crisis and myocarditis associated with anti-programmed cell death-1 (sintilimab) therapy for lung adenocarcinoma. *Ann Transl Med* 8: 250. doi: 10.21037/atm.2020.01.79

Yanase, T., Moritoki, Y., Kondo, H., Ueyama, D., Akita, H., and Yasui, T. (2021). Myocarditis and myasthenia gravis by combined nivolumab and ipilimumab immunotherapy for renal cell carcinoma: A case report of successful management. *Urol Case Rep* 34: 101508. doi: 10.1016/j.eucr.2020.101508

Yang, Y., Xu, L., Wang, D., Hui, B., Li, X., Zhou, Y., et al. (2021). Anti-PD-1 and regorafenib induce severe multisystem adverse events in microsatellite stability metastatic colorectal cancer: a case report. *Immunotherapy* 13: 1317-1323. doi: 10.2217/imt-2020-0327

Ying, Y.C., Tang, Q., Yang, K.W., Mi, Y., Fan, Y., Yu, W., et al. (2022). Clinical features of immune checkpoint inhibitor-related myositis in patients with urological cancer. *Beijing Da Xue Xue Bao Yi Xue Ban* 54: 644-651. doi: 10.19723/j.issn.1671-167X.2022.04.010

Zhang, B., Gyawali, L., Liu, Z., Du, H., and Yin, Y. (2022). Camrelizumab-Related Lethal Arrhythmias and Myasthenic Crisis in a Patient with Metastatic Thymoma. *Case Rep Cardiol* 2022: 4042909. doi: 10.1155/2022/4042909
